# Branch Unit Distribution Matters for Gene Delivery

**DOI:** 10.1021/acsmacrolett.3c00152

**Published:** 2023-05-23

**Authors:** Yinghao Li, Zhonglei He, Xianqing Wang, Zishan Li, Melissa Johnson, Ruth Foley, A. Sigen, Jing Lyu, Wenxin Wang

**Affiliations:** † Charles Institute of Dermatology, School of Medicine, University College Dublin, Dublin 4, Ireland, D04 V1W8; ‡ Branca Bunús Ltd, NovaUCD Belfield Innovation Centre, Dublin 4, Ireland, D04 V1W8

## Abstract

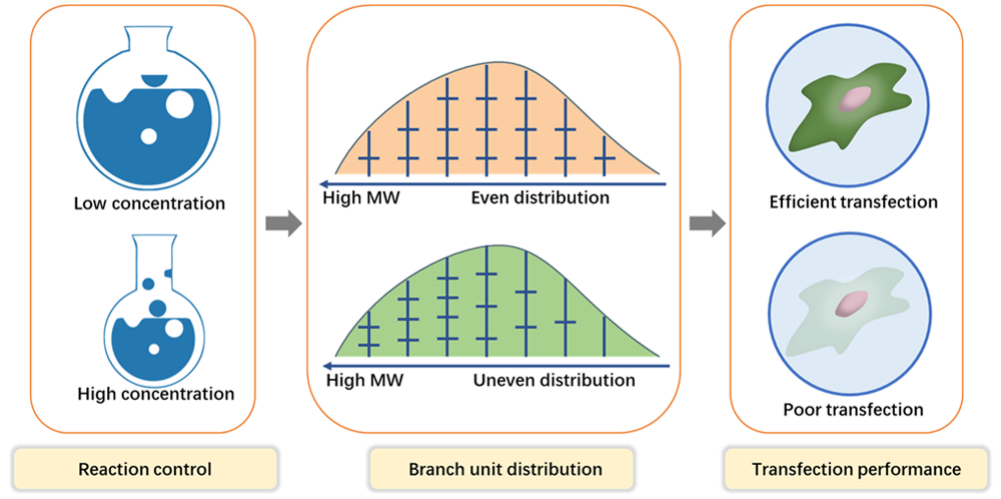

As a key nonviral gene therapy vector, poly(β-amino ester) (PAE) has demonstrated great potential for clinical application after two decades of development. However, even after extensive efforts in structural optimizations, including screening chemical composition, molecular weight (MW), terminal groups, and topology, their DNA delivery efficiency still lags behind that of viral vectors. To break through this bottleneck, in this work, a thorough investigation of highly branched PAEs (HPAEs) was conducted to correlate their fundamental internal structure with their gene transfection performance. We show that an essential structural factor, branch unit distribution (BUD), plays an important role for HPAE transfection capability and that HPAEs with a more uniform distribution of branch units display better transfection efficacy. By optimizing BUD, a high-efficiency HPAE that surpasses well-known commercial reagents (e.g., Lipofectamine 3000 (Lipo3000), jetPEI, and Xfect) can be generated. This work opens an avenue for the structural control and molecular design of high-performance PAE gene delivery vectors.

Gene therapy has become an essential field of advanced medical treatment. During the recent Covid-19 outbreak, nucleic acid vaccines from Pfizer, Moderna, Johnson & Johnson, AstraZeneca, etc., were mass-produced and supplied worldwide, saving hundreds of millions of lives.^1^ However, although gene therapy has created an enormous market and impressive clinical performance, highly efficient gene delivery vectors, the key to successful treatment, are still insufficient to meet the requirements of various applications and scenarios.^2,3^ Current widely used vectors can be divided into three groups: viral vectors, liposome vectors, and polymer vectors.^4−7^ Viral vectors are highly effective vehicles; however, significant safety concerns such as severe immune responses, activation of viral components, limited cargo therapeutic gene size, and expensive production have compromised their applications.^8^ As for liposome vectors, the lipoplexes suffer from poor colloidal stability in physiological environments and a potential inflammatory response.^9,10^ In comparison, the polymer vectors have the merits of high cargo capacity, biosafety, easy production, high stability, and modification, indicating a promising candidate for gene delivery vector development.

As one of the notable polymer vectors, poly(β-amino ester) (PAE) was first developed and applied as a transfection reagent by Langer’s group in 2000.^11^ During the past two decades it has emerged rapidly in gene delivery with the advantages of biodegradability, easy synthesis and modification, and high gene delivery efficiency.^5,12^ In 2016, highly branched PAEs were synthesized by Wang et al. (HPAEs) via a one-pot “A2 + B3 + C2”-type Michael addition approach.^13^ It was found that the branched structure significantly enhanced the transfection efficiency compared to the corresponding linear PAE (LPAE) and commercial transfection reagents. Additionally, different optimization strategies, for example, molecular designs (e.g., distribution precipitation) and a precise synthesis (e.g., the addition of cell-penetrating peptides), were applied to efficient PAE development.^14−16^ So far, thousands of PAE vectors have been synthesized and developed for clinical applications.^5,17,18^ However, their transfection performance is still not comparable to that of viral vectors.^5,12,19^ The gene delivery behavior of polymer vectors is closely related to their chemical structure, thus, to pursue the aim of improving the gene delivery performance of PAEs, the structural properties of PAEs have been continuously studied and optimized. While, through the studies during the past few decades, all the common structural factors of PAEs (e.g., chemical composition, molecular weight (MW) and topology etc.) have been explored so far,^13,20−22^ it seems that the optimization of the structure of PAEs has reached its limit. Does this mean that the optimization of PAEs has come to an end?

To answer this question, it is necessary to go back and carefully consider the fundamental synthesis and structure of PAEs to identify whether there are any critical structural characteristics that affect the polymer transfection efficiency but have not been realized. In this work, on the basis of the current most promising HPAE, a new structural factor, branch unit distribution (BUD), was for the first time found to impact the gene transfection performance. The correlation between the BUDs of HPAE and their gene transfection performance was investigated by comparatively analyzing the behavior of different HPAEs with different BUDs during the key steps of gene transfection. The results from this work provide a new principal for the future development of highly efficient transfection vectors from the perspective of polymer structural control.

The structure of HPAE is directly related to its synthesis conditions. To explore the relationship between the HPAE structure and the transfection performance, three batches of HPAEs were synthesized by copolymerizing 5-amino-1-pentanol (S5, A2 type monomer), pentaerythritol tetraacrylate (PTTA, B4 type monomer), and 1,4-butanediol diacrylate (BDA, C2 type monomer) via a facile one-pot “A2 + B4 + C2”-type Michael addition approach^13^ (Figure 1A) at varied concentrations (80%, 40%, and 30% w/v, Table 1). 1-(3-Aminopropyl)-4-methylpiperazine (E7) was added to end-cap these polymers, thus generating cationic-rich HPAEs named HPAE-A1, HPAE-B1, and HPAE-C1, respectively (Table 1). The functional monomers, BDA and S5 and the end group E7, were selected since they have been demonstrated to be effective components in the high-efficiency HPAE vectors.^13,23^ The tetraacrylate PTTA was employed as a branching monomer to create the highly branched structure. The chemical structures of HPAE-A1, HPAE-B1, and HPAE-C1 were characterized using gel permeation chromatography (GPC) and proton nuclear magnetic resonance spectroscopy (^1^H NMR). Figure 1B and Table 1 show that HPAE-A1, HPAE-B1, and HPAE-C1 have similar *M*_w,GPC_ values of 15.3, 12.2, and 13.2 kDa with the same dispersity (*Đ*) of 2.6, respectively. The Mark–Houwink exponent alpha (α) values of HPAE-A1, HPAE-B1, and HPAE-C1 are 0.26, 0.27, and 0.28, respectively (Table 1), which are much lower than the value for LPAE (α > 0.5),^24^ indicating the typical highly branched structure. The compositions of HPAE-A1, HPAE-B1, and HPAE-C1 were evaluated by ^1^H NMR analysis. As displayed in Figure 1C, the ratios of the integral area of methylene groups (b, g) on BDA units to that of methylene groups (h) on PTTA units show these three HPAE polymers have almost the same branching degree (BD).

**Figure 1:**
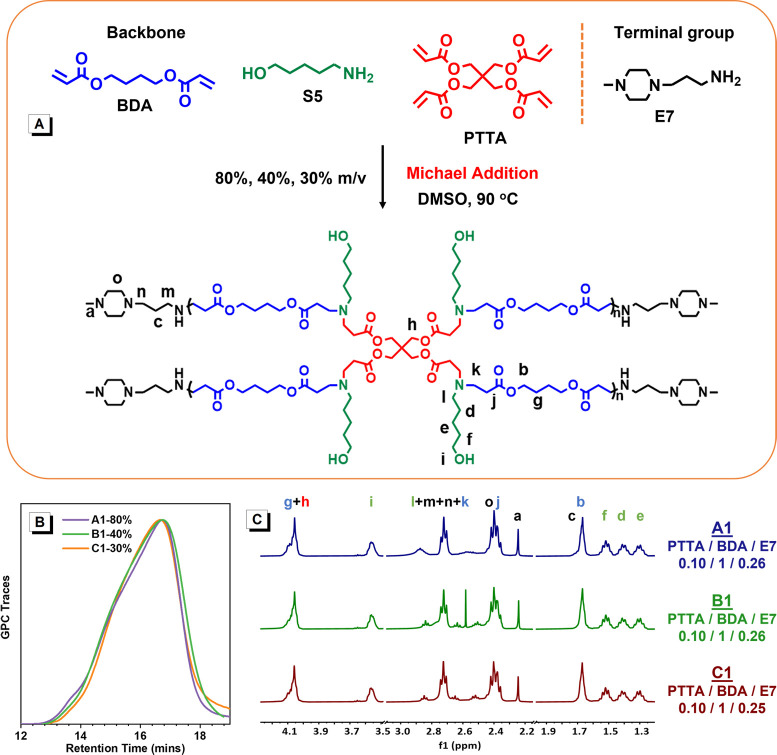
HPAE synthesis and structure characterization. (A) 5-Amino-1-pentanol (S5, A2), pentaerythritol tetraacrylate (PTTA, B4), and 1,4-butanediol diacrylate (BDA, C2) are first copolymerized via the “A2 + B4 + C2”-Michael addition and then end-capped with 1-(3-aminopropyl)-4-methylpiperazine (E7) to generate HPAE-A1, HPAE-B1, and HPAE-C1 at different reaction concentrations (A1, 80% w/v; B1, 40% w/v; C1, 30% w/v). (B) GPC traces of HPAE A1 to C1. HPAE-A1, HPAE-B1, and HPAE-C1 have similar *M*_w,GPC_, 15.3, 12.2, and 13.2 kDa, respectively, and the same dispersity (*Đ*), 2.6. (C) Comparison of the monomer and terminal group ratios of HPAE-A1, HPAE-B1, and HPAE-C1 utilizing the ^1^H NMR spectra. The three HPAE polymers have similar PTTA, BDA, and E7 contents.

**Table 1:** Reaction Conditions and Structure Characterization Results of HPAE Polymers

				molecular structure information			
polymer	reaction condition concn (w/v, %)	feed ratio [PTTA]:[BDA]^a^	composition ratio [PTTA]:[BDA]^b^	*M*_w,GPC_^c^ (Da)	*M*_n,GPC_^c^ (Da)	*Đ*^c^	α^d^
HPAE-A1	80	0.1:1	0.1:1	15268	5686	2.6	0.26
HPAE-B1	40	0.1:1	0.1:1	12202	4650	2.6	0.27
HPAE-C1	30	0.1:1	0.1:1	13179	4962	2.6	0.28

a Reaction: DMSO as solvent, 90 °C; end-capping: E7.

b Calculated from ^1^H NMR spectra.

c Determined by GPC with RI detector.

d The Mark–Houwink exponent α value.

Subsequently, HPAE-A1, HPAE-B1, and HPAE-C1 were applied to the in vitro analysis to assess their transfection capability. The efficiency of these three HPAE vectors in gene transfection was evaluated by examining the cytoplasmic green fluorescent protein (GFP) expression. Well studied commercial transfection reagents, Lipo3000, jetPEI, and Xfect, were used as positive controls, providing a good benchmarking for comparison. The human-derived embryonic kidney cells (HEK-293) and a disease model, recessive dystrophic epidermolysis bullosa keratinocytes (RDEBK), were used for in vitro assessment. Figure 2A,B outlines the GFP-encoding plasmid transfection results of different cell lines after transfection with HPAE-A1, HPAE-B1, and HPAE-C1. Surprisingly, transfection with both cell types shows that HPAE-C1 (synthesized at a lower concentration (30% w/v)) brought about higher GFP expression than HPAE-A1 and HPAE-B1 and maintained high cell viability (Figures S1 and S2). Moreover, as shown by flow cytometry (Figures 2C and S3), the percentage of GFP-positive RDEBK cells achieved by HPAE-A1 and HPAE-B1 polyplexes is only 37% and 64% at the polymer/DNA weight ratio (w/w) of 20:1, respectively. In contrast, HPAE-C1 polyplexes achieved a much higher level of GFP-positive cells, 77% and 89% at w/w ratios of 20:1 and 30:1 respectively, which is also greater than that of commercial reagents (38% of Lipo3000, 57% of jetPEI, and 79% of Xfect). In addition, the median fluorescence intensity (MFI) of RDEBK cells transfected by HPAE-C1 polyplexes at a w/w ratio of 30:1 is higher than that transfected by HPAE-A1, HPAE-B1, and the commercial reagent controls.

**Figure 2:**
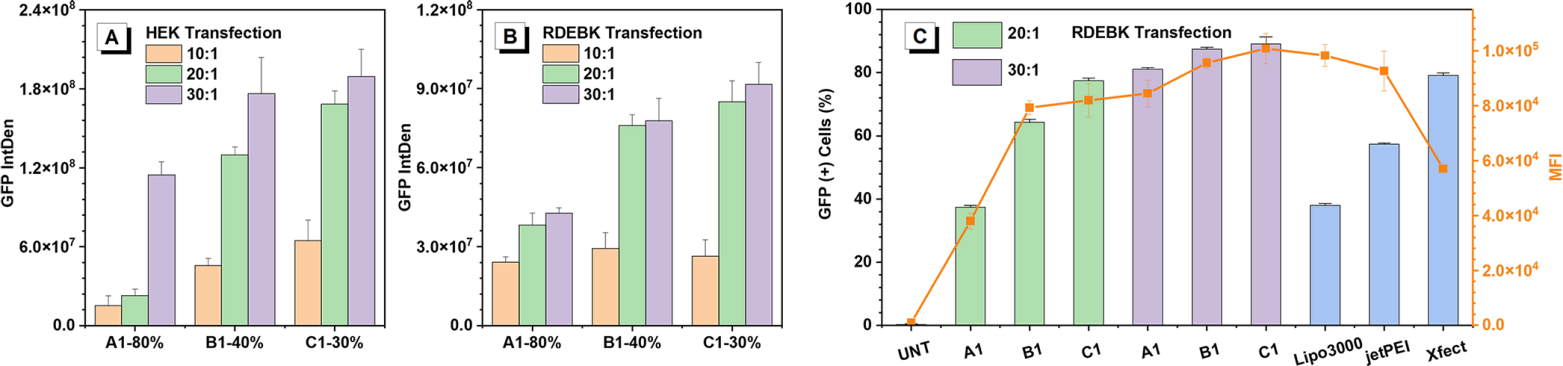
Comparison of the gene transfection efficiency of HPAE-A1, HPAE-B1, and HPAE-C1. GFP expression of (A) HEK 293 cells and (B) RDEBK cells 48 h post-transfection. (C) Percentage of GFP-positive RDEBKs and the mean fluorescence intensity (MFI) of cells after transfection. Untreated (UNT) cells were used as the negative control.

To further confirm and analyze the mechanism behind the above variations in HPAE gene transfection performance, HPAE-A1, B1, and C1 were carefully studied in terms of several key steps in gene transfection: DNA binding and condensation, serum stability, polyplex cellular uptake, and DNA protection.^25^ The first step for successful gene delivery is that vectors can effectively package DNA to form nanosized polyplexes. The interaction of HPAE-A1, HPAE-B2, and HPAE-C3 with DNA was analyzed by a PicoGreen assay, dynamic light scattering (DLS), and transmission electron microscopy (TEM). As shown in Figure 3A, over the range of tested polymer/DNA weight ratios (w/w, from 10:1 to 30:1), all three HPAEs can achieve over 80% DNA binding and condense DNA into polyplexes with a size of around 200 nm (Figure S4), indicating the strong electrostatic interaction of three HPAE vectors with DNA. Then, the serum stability of the polyplexes of HPAE-A1, HPAE-B1, and HPAE-C1 was tested. Given that DNA uptake is a continuous process and there is interference of serum proteins in the physiological environment, polyplex stability is an essential factor that needs to be considered for efficient gene delivery. Figure 3B shows the ratio of the polyplex size of HPAE-A1, HPAE-B1, and HPAE-C1 after 4 h postincubation in serum to that at 0 h. The results demonstrate that the polyplex size of HPAE-A1 increased nearly 3-fold within 4 h, while HPAE-B1 and HPAE-C1 are more stable. In particular, at the polymer/DNA weight ratios (w/w) of 20:1 and 30:1, the size of polyplex HPAE-C1 was almost unchanged after 4 h postincubation, exhibiting good stability in serum. With the strong serum stability, the cellular uptake of the HPAE-C1/DNA polyplex was almost twice that of HPAE-A1/DNA (Figure 3C, measured by the fluorescence of Cy3-labeled DNA of HEK 293 cells). In addition, regarding the DNA protection ability under acidic conditions, HPAE-C1 protected DNA better than HPAE-A1 and HPAE-B1 by maintaining over 80% DNA binding (at a polymer/DNA weight ratio of 30:1) after 4 h incubation at 37 °C (Figure 3D).

**Figure 3:**
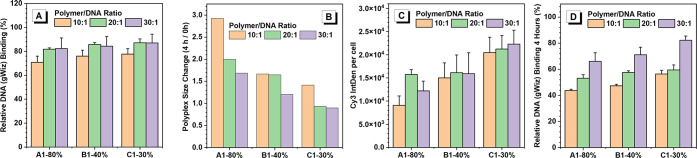
In vitro investigation of the gene transfection performance of HPAE-A1, HPAE-B1, and HPAE-C1 during the key steps in gene transfection. (A) Assessment of the DNA binding capability of HPAE-A1–C1 vectors at various polymer/DNA weight ratios (w/w) using the PicoGreen assay. (B) Serum stability of HPAE-A1–C1 polyplexes, assessed by the ratio of the average polyplex size after 4 h postincubation in media with 10% FBS at 37 °C to that at 0 h. The polyplexes size evaluation at 0 and 4 h was repeated in triplicate. (C) Cellular uptake of HPAE-A1–C1 polyplexes, evaluated by the fluorescence of the Cy3-labeled DNA of HEK 293 cells 4 h post-transfection. (D) DNA protection capability of HPAE-A1–C1 under acidic conditions (25 mM sodium acetate), evaluated by DNA binding efficiency after 4 h incubation at 37 °C using a PicoGreen assay.

The above results demonstrated that compared to HPAE-A1 and HPAE-B1, HPAE-C1 contributed more to the polyplex stability and to DNA protection, which are favorable for cellular uptake, thus ultimately promoting higher gene transfection efficiency. However, these results contradict our conventional understanding that polymer vectors that have similar topology, composition, MWs, *Đ*, and BD exhibit similar transfection performance. Therefore, we considered whether there were other critical structure factors, beyond those that are commonly evaluated, including topology, composition, MWs, *Đ*, BD, etc., that could cause the different transfection performances of HPAE-A1, HPAE-B1, and HPAE-C1. To clarify this, further study is required to explore more subtle structural differences between HPAE-A1, HPAE-B1, and HPAE-C1, the findings from which may open a new avenue for the PAE vector design.

As mentioned before, minimal changes in the polymer structure could have an important impact on transfection efficiency. Therefore, it is conceivable that the variations in the HPAE structure due to the different synthesis concentrations could be responsible for their different transfection properties. The synthesis strategy of HPAE is to introduce a multifunctional monomer “Bn” to the step growth polymerization (SGP) process.^26−28^ The introduction of “Bn” not only brings more terminal groups, but also changes the configuration of the macromolecule from a 2D linear to a 3D branched structure. The more “Bn” present, the more pronounced this transition. Since HPAE is composed of a group of macromolecules of different sizes (*Đ* > 2),^13^ where the structure of the multifunctional monomer and bifunctional monomer are randomly distributed, the distribution of the branching unit “Bn” on different macromolecular chains may vary depending on the synthesis method. This inspired us to hypothesize that, besides the common properties (MW, dispersity, average composition ratio, etc.) of the entire HPAE polymer, the branch unit distribution in HPAE could play a role for gene delivery efficiency.

With this question in mind, HPAE-A1, HPAE-B1, and HPAE-C1 were fractionated to obtain a series of polymer components within different ranges of MWs, HPAE-AS1–AS5, HPAE-BS1–BS5, and HPAE-CS1–CS5, respectively, via elution fractionation. As depicted in Figure 4A, taking HPAE-A1 as an example, HPAE-A1 (also named as HPAE-AP1 in Figure 4A) was first dissolved in acetone to a concentration of 100 mg/mL, and the solution was slowly added into a solvent mixture of acetone/diethyl ether (v/v = 1/9) under gentle agitation at room temperature. The precipitate was then collected as a residue polymer HPAE-AP2, and the component HPAE-AS1 was obtained by evaporating the remaining supernatant solution. A similar process for HPAE-AP2 to HPAE-AP5 produced polymer components HPAE-AS2 to HPAE-AS5 by using solvent mixtures with increasing acetone content (acetone/diethyl ether = 2/8 to 4/6; Figure 4A). Here, HPAE-AP5 was also named as HPAE-AS5, representing the highest molecular weight components of HPAE-A1. The polymer components of HPAE-BS1–BS5, and HPAE-CS1–CS5 were obtained from HPAE-B1 and HPAE-C1, respectively, following the same process as described above.

**Figure 4:**
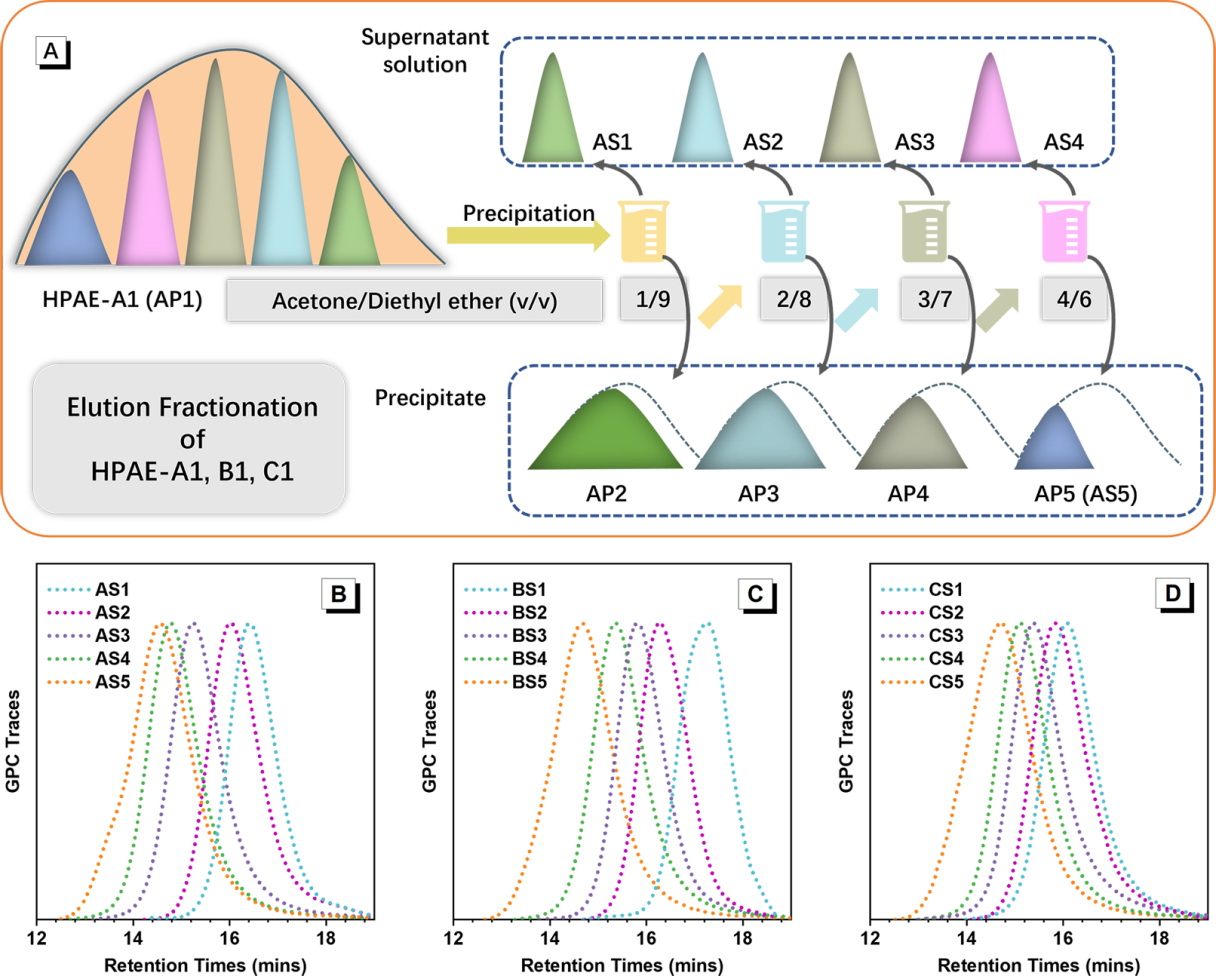
HPAE fractionation process and the characterization of different polymer components. (A) HPAE-A1, HPAE-B1, and HPAE-C1 were fractionated into HPAE-AS1–AS5, HPAE-BS1–BS5, and HPAE-CS1–CS5, respectively, by precipitating into a solvent mixture of acetone/diethyl ether. The components HPAE-AS1–AS5, HPAE-BS1–BS5, HPAE-CS1–CS5 were collected from the supernatant solution. The residual polymers HPAE-AP2–AP5, HPAE-BP2–BP5, and HPAE-CP2–CP5 were collected from the precipitates. HPAE-AP4, HPAE-BP4, and HPAE-CP4 were also named as HPAE-AS5, HPAE-BS5, and HPAE-CS5, respectively, which represents the component with the highest molecular weight. GPC traces are presented of polymer components (B) HPAE-AS1–AS5, (C) HPAE-BS1–BS5, and (D) HPAE-CS1–CS5.

Figure 4B–D and Table S1 show the GPC and ^1^H NMR characterization results for each individual component HPAE-AS1–AS5, HPAE-BS1–BS5, and HPAE-CS1–CS5 fractionated from HPAE-A1, HPAE-B1, and HPAE-C1 respectively. These data clearly illustrate the parallel movements of the molecular weights from low to high, *M*_w,GPC_ = 4542–30525 Da for HPAE-AS1–AS5; *M*_w,GPC_ = 2444–26535 Da for HPAE-BS1–BS5; *M*_w,GPC_ = 6321–27130 Da for HPAE-CS1–CS5.

Then, using a method similar to Figure 1C (to calculate the average compositions of HPAE-A1, HPAE-B1, and HPAE-C1 by ^1^H NMR), the ratios of PTTA units to BDA units on each polymer component fractionated from HPAE-A1, HPAE-B1, and HPAE-C1 were calculated here to evaluate the BUD, i.e., PTTA distribution in HPAE-A1, HPAE-B1, and HPAE-C1 (Table S1 and Figures 5 and S5–S7). Figure 5 outlines the distribution of the PTTA units in different MW intervals for HPAE-A1, HPAE-B1, and HPAE-C1. Remarkably, it can be observed that the composition distribution of PTTA in HPAE-C1 was more uniform compared to that in HPAE-B1 and HPAE-C1. Specifically, in HPAE-C1, which was synthesized at a lower reaction concentration, the molar ratio of PTTA/BDA varied within a relatively narrower range among polymer components of different MWs. However, for HPAE-A1 and HPAE-B1 which were obtained at higher concentrations, their PTTA/BDA molar ratio varied within a broader range. Compared to HPAE-C1, the low MW components in HPAE-A1 and B1 contained less PTTA, while in contrast, the high MW components of HPAE-A1 and HPAE-B1 contained a higher proportion of PTTA, resulting in a broader distribution of branch units. This trend became more pronounced as the synthesis concentration increased.

**Figure 5:**
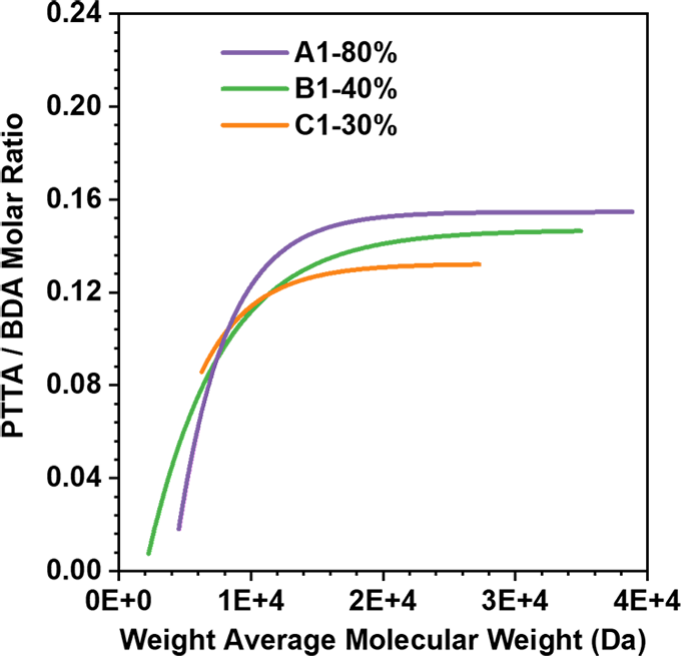
Fitted branch unit distribution (i.e., molar ratio of PTTA to BDA at different MWs) of HPAE-A1–C1, calculated based on the ^1^H NMR spectra of different MW components (Figures S5–S7).

Based on the above BUD characterization, it is understandable why HPAE-C1 exhibited better transfection performance compared to HPAE-A1 and HPAE-B1, though all general structural information was similar. On the one hand, the higher ratio of PTTA units in the low MW components of HPAE-C1 brought more terminal groups, which contributed to the shielding of the DNA charges, regular coil folding, and DNA compression. On the other hand, the lower content of PTTA in the high MW components of HPAE-C1 enabled it to form a more flexible high-MW structure, which could wrap more tightly around the polyplex, resulting in the superior stability of the nanoparticle structure. Therefore, the HPAE-C1/DNA polyplex with its better capacity for DNA binding, protection, and serum stability is more favorable for cellular uptake, thus improving the gene transfection efficiency. According to the above studies, the BUD of HPAE, which has not been evaluated in previous HPAE vector designs, has an important effect on gene delivery performance.

In this work it is reported that the HPAE vector synthesized at a lower reaction concentration achieved better gene delivery performance compared to the HPAE vectors synthesized at higher reaction conditions with similar MWs, *Đ*, and BD. Inspired by this, a thorough investigation of the subtle structural characteristics of the HPAEs was conducted and correlated with their behavior during the key steps of gene transfection. We show here for the first time that BUD acts as an essential structural factor for HPAE transfection capability. It is demonstrated that HPAE, with a more uniform distribution of branch units (i.e., a higher ratio of branch units within the low MW components and a lower ratio within the high MW components), maintains better transfection efficacy. This provides valuable insights into the structural control and future development of high-performance polymer gene delivery vectors.

## Supporting Information Available

Materials; Synthesis and characterization methods; Cytotoxicity study in HEK and RDEBK cells; Flow cytometry gating; TEM characterization of polyplexes; ^1^H NMR spectra of HPAE-A1, HPAE-B1, and HPAE-C1; Table of GPC and ^1^H NMR analysis results of different HPAE components (PDF)
