# Supplementary material for: Branch Unit Distribution Matters for Gene Delivery
Source: ACS Macro Lett. 2023 May 23;12(6):780–6. doi: 10.1021/acsmacrolett.3c00152 (PMC10286303; doi:10.1021/acsmacrolett.3c00152)
Supplement: Supplementary file 1 — mz3c00152_si_001.pdf [file mz3c00152_si_001.pdf]

Supporting Information

Branch Unit Distribution Matters for Gene Delivery

Yinghao Li,[†] *Zhonglei He,*[†] *Xianqing Wang,*[†] *Zishan Li,*[†] *Melissa Johnson,*[†] *Ruth Foley,*[†,‡] *Sigen A,*[†] *Jing Lyu,*[*,†] *and Wenxin Wang*[*†]

[†]Charles Institute of Dermatology, School of Medicine, University College Dublin, Dublin 4, Ireland

[†]Branca Bunús Ltd, NovaUCD Belfield Innovation Centre, Ireland

1. Materials

1,4-butanediol diacrylate (BDA), 5-amino-1-pentanol (S5), and pentaerythritol tetraacrylate (PTTA) were purchased from Sigma-Aldrich. 1-(3-aminopropyl)-4-methylpiperazine (E7) was purchased from Fisher Scientific. Lithium bromide (LiBr) for GPC measurements was purchased from Sigma-Aldrich. Dimethyl sulfoxide (DMSO), dimethylformamide (DMF), acetone, and diethyl ether were purchased from Fisher Scientific. Deuterated chloroform ($CDCl_3$) was purchased from Sigma-Aldrich. Hank's balanced salt solution and alamarBlue Assay Kit were purchased from Sigma and Invitrogen. Sodium acetate (Sigma) was diluted to 0.025 M before use. PicoGreen was purchased from Life Technologies. Cell culture Dulbecco's modified Eagle Medium (DMEM) was purchased from Sigma. Fetal bovine serum (FBS, Gibco, was filtered through 0.2 μm filters before use. The gWiz-GFP commercial plasmid was obtained from Aldevron, Fargo, ND, USA. Xfect was purchased from Medical Supply Co. Ltd. Lipofectamine 3000 (Lipo3000) transfection reagent was purchased from Bio-sci. JetPEI was purchased from Polyplus Transfection, Illkirch-Graffenstaden, Strasbourg, France.

2. Synthesis and characterization methods

HPAE polymer synthesis

Branched-PAEs (HPAEs) were synthesized through a facile Michael addition reaction. Monomer feed ratios and reaction conditions for the synthesis of HPAE base polymers are listed in Table 1. Taking the synthesis of HPAE-A1 as an example, BDA (3.96 g), S5 (2.06 g), and PTTA (0.70 g) were dissolved in DMSO (1.53 mL). Then, the solution

was bubbled under argon for 15 mins to remove oxygen. After that, the reaction mixture was merged into the preheated oil bath and react with stirring at 90 °C for the desired time. Agilent 1260 Infinite gel permeation chromatography (GPC) and nuclear magnetic resonance (NMR) were used to monitor the reaction. The reaction was stopped by diluting the mixture to 100 mg/mL with DMSO when M_w was approaching 10 kDa. E7 (2.51 g) was then added to end-cap the acrylate-terminated base polymer at room temperature for 48 h. After that, HPAE polymers were precipitated into diethyl ether three times for purification and dried under vacuum before being stored at −20 °C.

Elution fractionation

After generating polymers HPAE-A1, HPAE-B1, and HPAE-C1, they were franctionated to obtain different components with a range of molecular weights (HPAE-AS1 to AS5, HPAE-BS1 to BS5, and HPAE-CS1 to CS5). Taking the HPAE-A1 as an example, the fractionation procedure is as follows: HPAE-A1 was dissolved in acetone to a concentration of 100 mg/mL, then the solution was slowly added into the mixed solvent of acetone and diethyl ether (v/v=1/9) under gentle agitation at room temperature. The solvent in the supernatant solution was removed by rotary evaporator and the product was collected as component HPAE-AS1. Then the precipitate was redissolved in acetone and precipitated into another mixed solvent with higher acetone ratio (acetone/diethyl ether = 2/8) to generate component HPAE-AS2. By repeating the stepwise precipitation process, Components HPAE-AS1 to AS5 were obtained. HPAE-BS1 to BS5 and HPAE-CS1 to CS5 were obtained following the same procedure.

Molecular weight and dispersity measurements

Number average molecular weight (M_n), weight average molecular weight (M_w), and dispersity ($Đ$) of polymers were determined by GPC equipped with a refractive index detector (RI), a viscometer detector (VS DP) and a dual angle light scattering detector (LS 15° and LS 90°). To sample the molecular weight of polymers during the polymerization process, 20 μL of the reaction mixture was collected at different time points, and diluted with 1 mL of DMF, then filtered through a 0.2 μm filter, and finally measured by GPC. The columns (PolarGel-M, Edinburgh, UK, 7.5 mm × 300 mm, two in series) were eluted with DMF and 0.1% LiBr at a flow rate of 1 mL/min at 60 °C. Columns were calibrated with linear poly(methyl methacrylate) (PMMA) standards.

Nuclear magnetic resonance (NMR)

The chemical structure and composition of polymers were confirmed with ^1H NMR. Polymer samples were dissolved in $CDCl_3$. Measurements were carried out on a Varian Inova 400 MHz spectrometer.

Polyplex preparation

Generally, the polymers were initially dissolved in DMSO to stock solutions (100 mg/mL), and the stock solutions were further diluted with 25 mM sodium acetate buffer according to the w/w ratio. DNA was diluted to 0.1 mg/mL with sodium acetate buffer. The polymer solutions were added into the DNA solution, vortexed for 10 s, and allowed to stand for 15 min.

PicoGreen assays

The polyplex was prepared as described above. 2 μg of DNA was used for each sample

preparation. 60 μL of PicoGreen solution was prepared according to the supplier's instructions, added to the DNA and allowed to incubate for another 5 min. Ina 96-well plate, 200 μL of medium (without serum) or water was added, and 30 μL of the polyplex solution was then added. Fluorescence measurements were carried out with a plate-reader with an excitation at 490 nm and emission at 535 nm. All the measurements were repeated in triplicate.

Size measurement of polyplexes

The polyplex was prepared as described above and their sizes were measured with a Malvern Panalytical Zetasizer (ZTS1240). All the measurements were repeated in triplicate.

Morphology measurement of polyplexes

Morphology of polyplexes was characterized by transmission electron microscopy (TEM). After preparing polyplexes as described above, the polyplex solution was dropped onto 200 mesh copper grids and allowed to dry for 20 mins. The polyplex was further washed with distilled water twice to remove excess salts before imaging. Images were captured on a FEI Tecnai 120 TEM at 120 kV in UCD Conway Imaging Core Center.

Cell culture

RDEBK cells were cultured using standard cell culture techniques in keratinocyte growth complete FAD medium (KCa). HEK 293 cells were cultured in Dulbecco's modified Eagle's medium (high glucose) containing 10% fetal bovine serum and 1% penicillin-s. Cells were cultured at 37 ^{o}C with 5% CO_2 in a humidified incubator under

standard cell culture techniques.

Flow Cytometric Analysis

After 48 h post-transfection, cells were harvested with trypsin-EDTA and washed with PBS, then re-suspended in PBS with 2% FBS. The flow cytometry measurements were carried out on an Accuri C6 system in triplicate. Data analysis was performed using the Cytexpert software.

Polyplex cellular uptake

GFP DNA was labelled with a Cy3 (a red fluorescent dye) labelling kit as per the recommended protocol. HEK cells were seeded in 96-well plates. Gene transfection was conducted as above with 0.5 μg of DNA per well. After 4 hours, the medium was removed, and cells were fixed with 4% paraformaldehyde after washing with PBS three times. Next, the cells were permeabilized with 0.1% Triton X-100 and stained with DAPI, followed by visualization under a fluorescence microscope (Olympus IX81). All the measurements were repeated in triplicate.

Cytotoxicity assessment (alamarBlue assay)

To perform the alamarBlue assay, cell supernatants were first removed, then cells were washed with HBSS, followed by adding 10% alamarBlue reagent in HBSS. Living, proliferating cells maintain a reducing environment within the cytosol of the cell, converting the non-fluorescent ingredient resazurin in alamarBlue to the highly fluorescent compound resorufin. This reduction results in a color change from blue to light red and allows for the quantitative measurement of cell viability based on the increase in overall fluorescence and color of the media. The alamarBlue solution from

each well was transferred to a fresh flat-bottomed 96-well plate for fluorescence measurements at 590 nm. Control cells without any treatment were used to normalize the fluorescence values and plotted as 100% viable. All the measurements were repeated in triplicate.

3. Experimental data

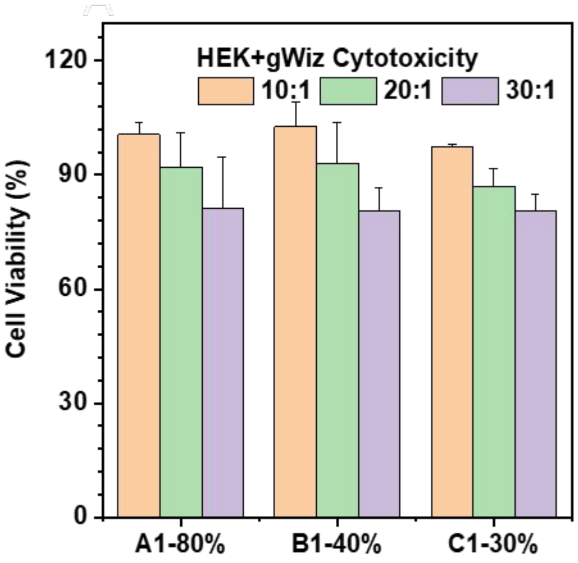

Figure S1. *In vitro* investigation of the cytotoxicity of HPAE-A1, HPAE-B1 and HPAE-C1. Cell viability of HEK 293 cells 48 h post-transfection.

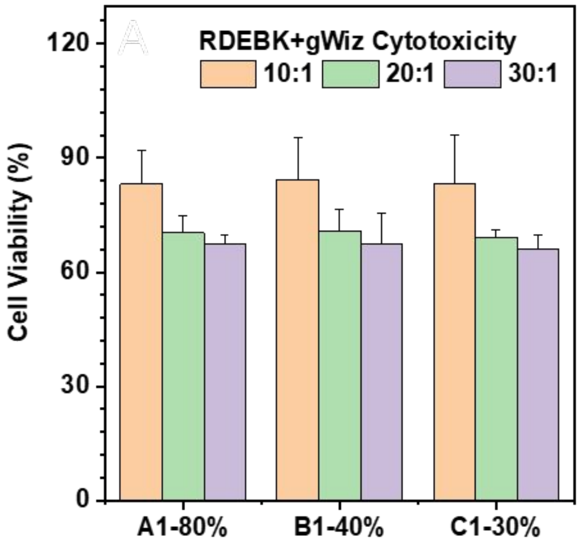

Figure S2. *In vitro* investigation of the cytotoxicity of HPAE-A1, HPAE-B1 and HPAE-C1. Cell viability of RDEBK cells 48 h post-transfection.

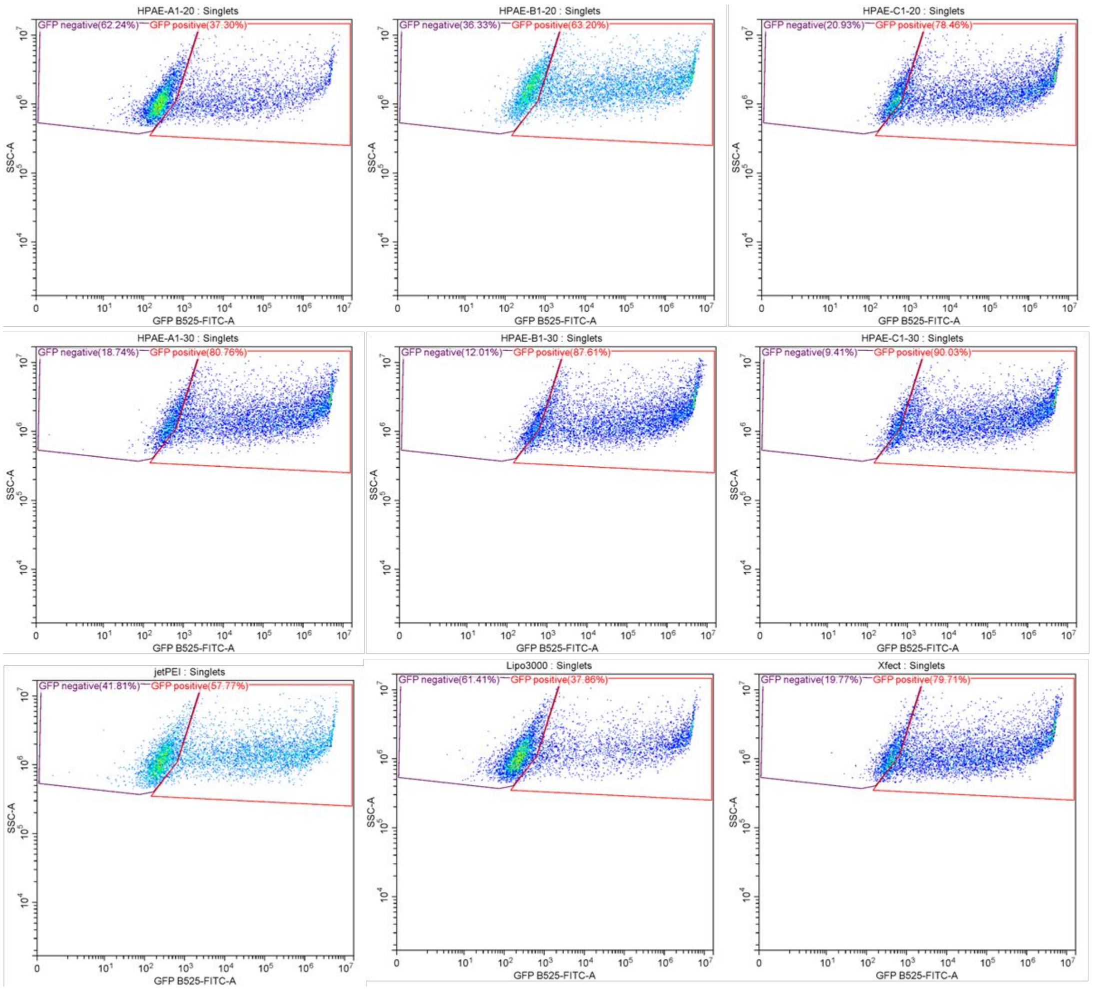

Figure S3. *In vitro* investigation of the gene transfection performance of HPAE-A1, HPAE-B1 and HPAE-C1. GFP-positive cells gating strategy for flow cytometry.

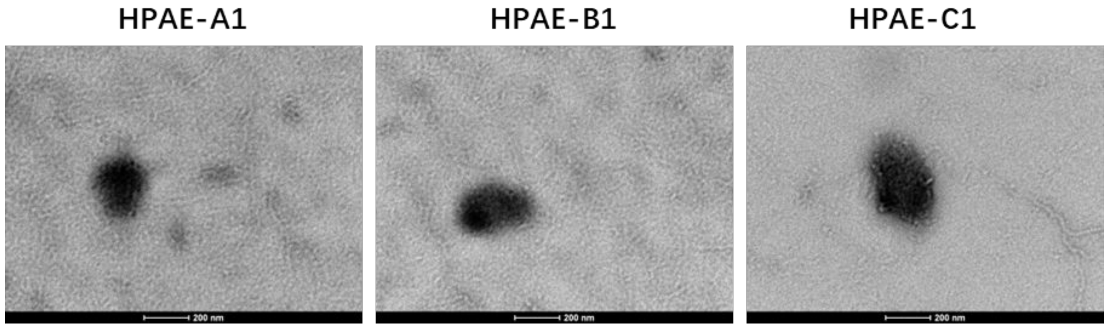

Figure S4. TEM image of the nanoscale polyplexes formed by HPAE-A1, HPAE-B1 and HPAE-C1 with DNA (the polymer/DNA weight ratio is 30:1).

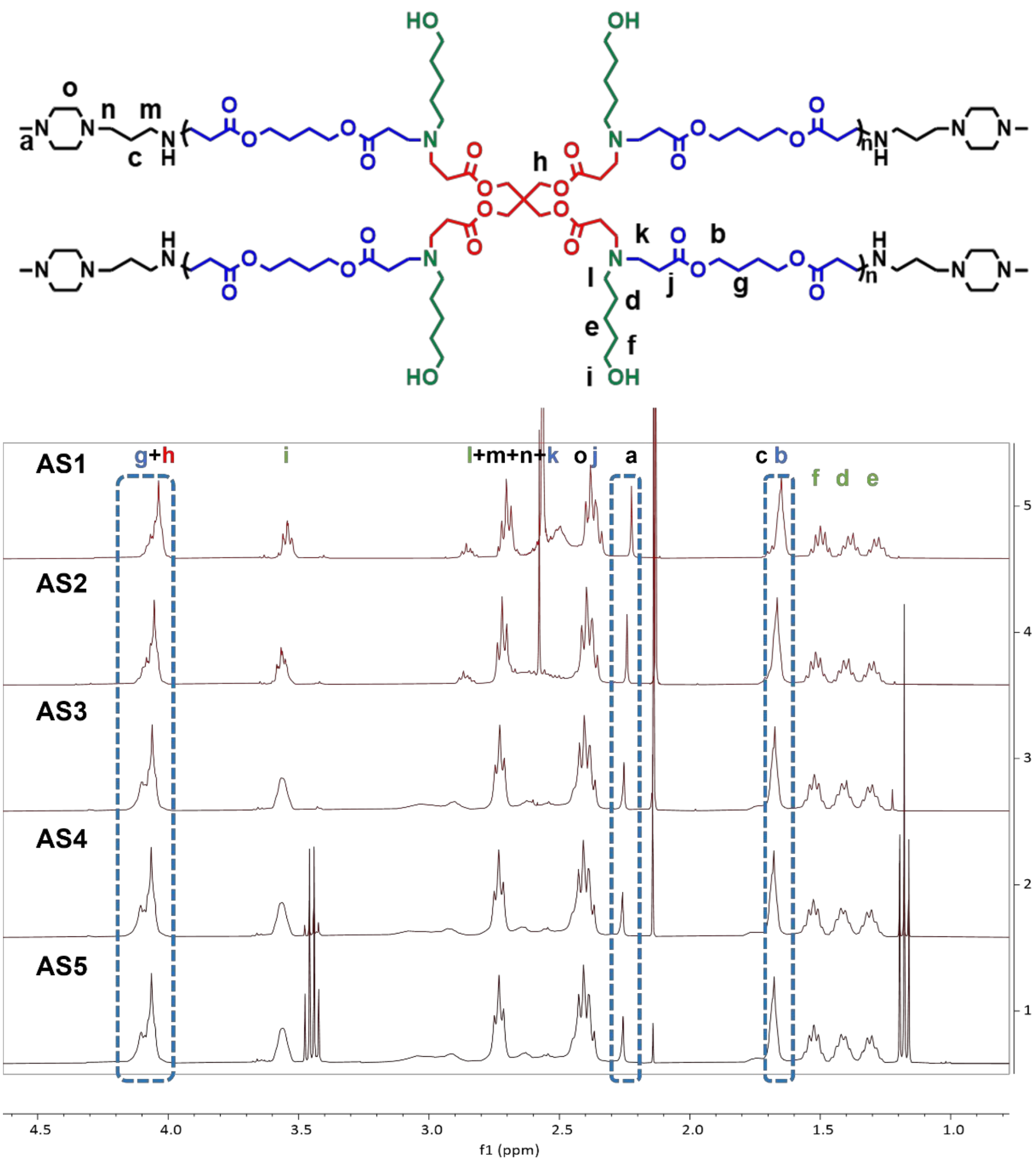

Figure S5. ^{1}H NMR spectra of the fractionation products HPAE-AS1 to AS5 of HPAE-A1.

Molar ratio calculation of [PTTA] / [BDA] / [E7] = $[(I_{g+h} - I_b) / 8] / [I_b / 4] / [I_a / 3]$

where I_{g+h}, I_b and I_a stand for the integral intensity of peak g+h, b and a in ^{1}H NMR spectra.

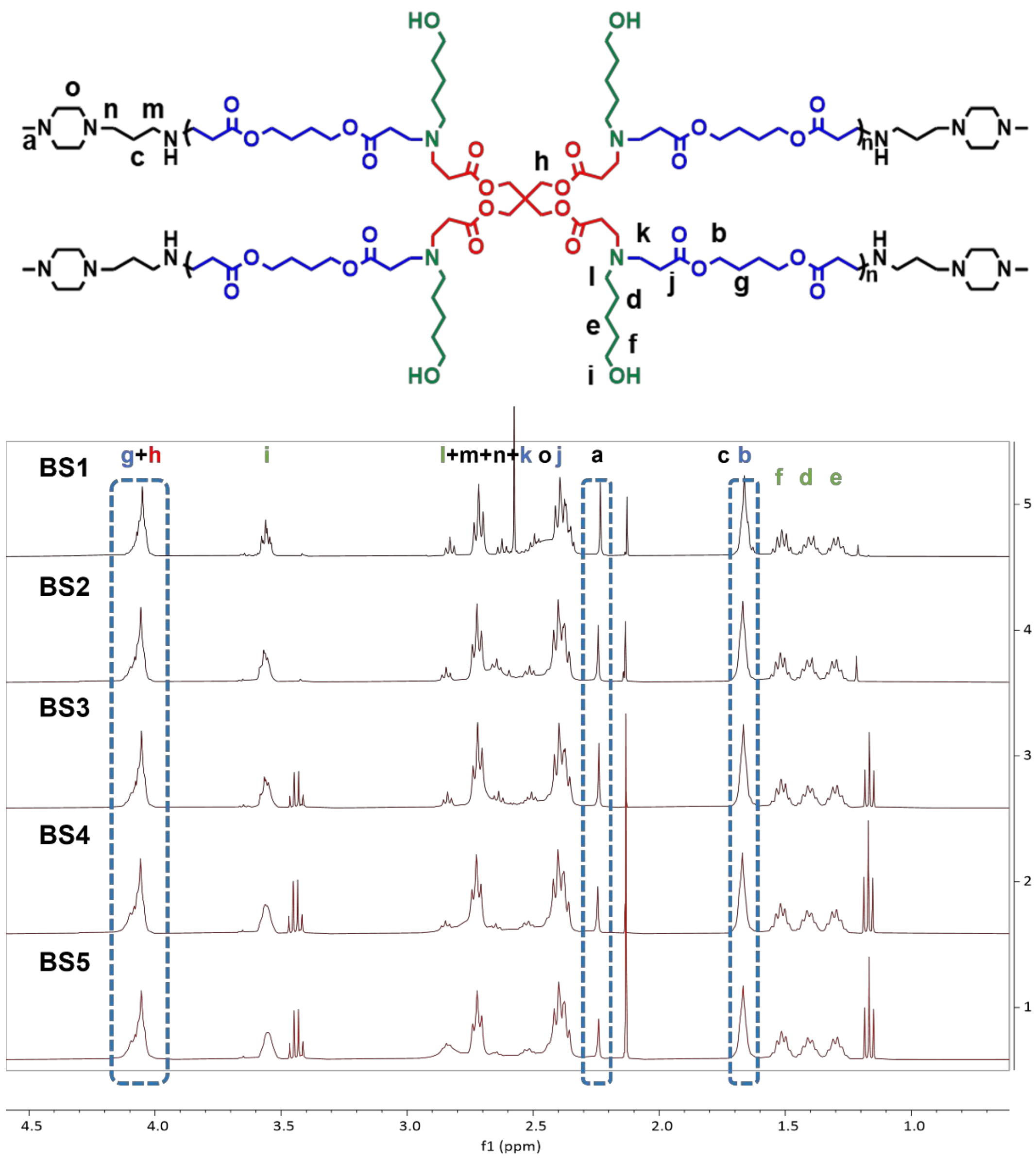

Figure S6. ^{1}H NMR spectra of the HPAE-B1 fractionation products.

Molar ratio calculation of [PTTA] / [BDA] / [E7] = $[(I_{g+h} - I_b) / 8] / [I_b / 4] / [I_a / 3]$

where I_{g+h}, I_b and I_a stand for the integral intensity of peak g+h, b and a in ^{1}H NMR spectra.

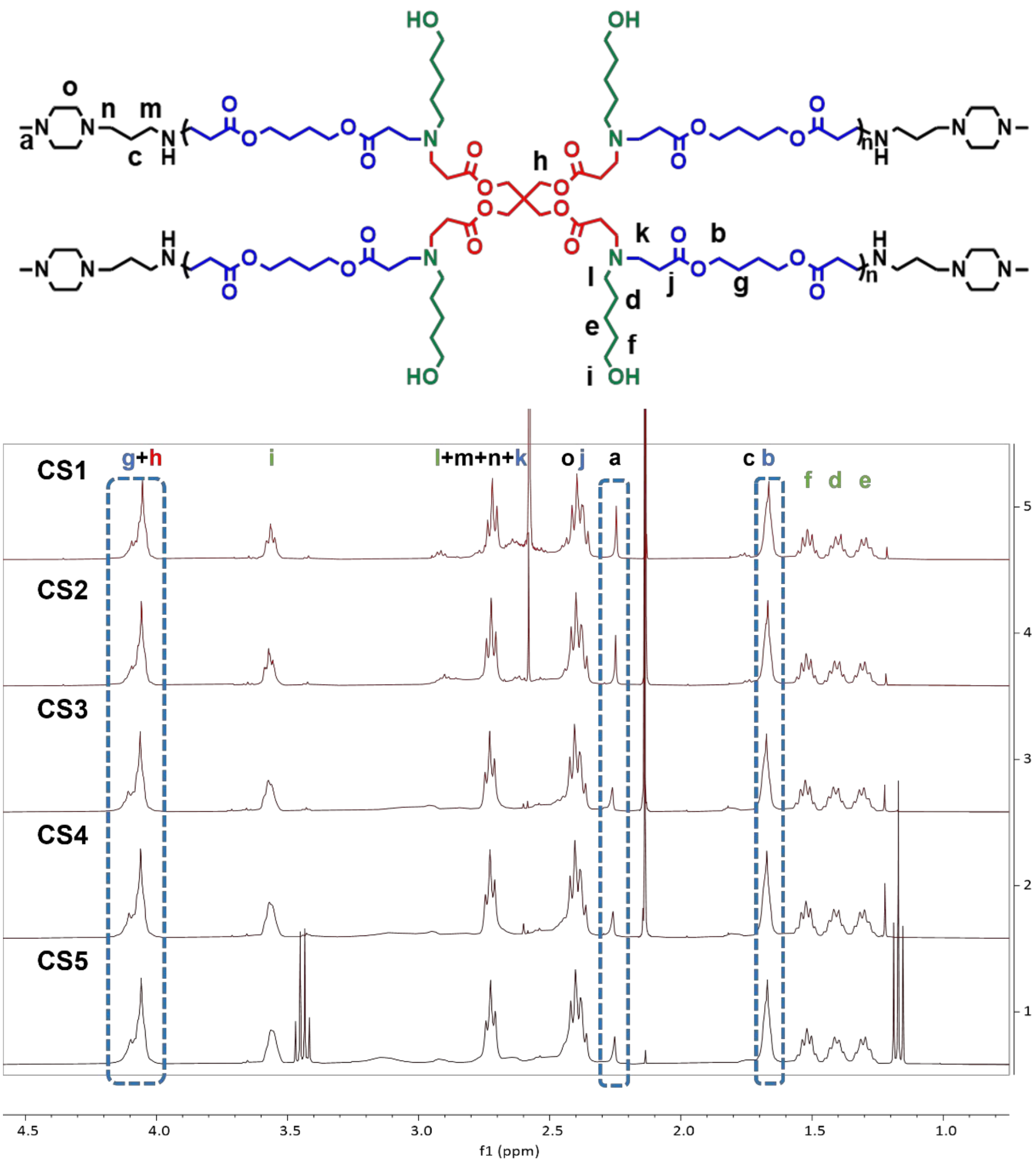

Figure S7. ^{1}H NMR spectra of the HPAE-C1 fractionation products.

Molar ratio calculation of [PTTA] / [BDA] / [E7] = $[(I_{g+h} - I_b) / 8] / [I_b / 4] / [I_a / 3]$

where I_{g+h}, I_b and I_a stand for the integral intensity of peak g+h, b and a in ^{1}H NMR spectra.

Table S1. GPC and ^{1}H NMR analysis results of different HPAE components obtained from fractionation

Polymer component	$M_{w,GPC}$ [a] (Da)	$M_{n,GPC}$ [a] (Da)	$Đ$ [a]	[PTTA]:[BDA] [b]
HPAE-AS1	4542	3263	1.39	0.018
HPAE-AS2	6389	4192	1.52	0.071
HPAE-AS3	13737	8171	1.68	0.142
HPAE-AS4	21310	12353	1.72	0.150
HPAE-AS5	30525	14797	2.06	0.154
HPAE-BS1	2444	1909	1.28	0.012
HPAE-BS2	5080	3889	1.30	0.063
HPAE-BS3	7996	5724	1.40	0.097
HPAE-BS4	12304	7128	1.72	0.124
HPAE-BS5	26535	13932	1.91	0.145
HPAE-CS1	6321	4205	1.50	0.085
HPAE-CS2	7593	4988	1.52	0.100
HPAE-CS3	11723	6447	1.82	0.121
HPAE-CS4	15571	9022	1.72	0.126
HPAE-CS5	27130	13860	1.96	0.133

[a] Determined by GPC RI detector. [b] [PTTA]:[BDA] was calculated from the molar ratio of PTTA and BDA according to ^{1}H NMR spectra.
